# Associations of *NOD2* polymorphisms with Erysipelotrichaceae in stool of in healthy first degree relatives of Crohn’s disease subjects

**DOI:** 10.1186/s12881-020-01115-w

**Published:** 2020-10-15

**Authors:** Williams Turpin, Larbi Bedrani, Osvaldo Espin-Garcia, Wei Xu, Mark S. Silverberg, Michelle I. Smith, Juan Antonio Raygoza Garay, Sun-Ho Lee, David S. Guttman, Anne Griffiths, Paul Moayyedi, Remo Panaccione, Hien Huynh, Hillary A. Steinhart, Guy Aumais, Levinus A. Dieleman, Dan Turner, CCC IBD GEM Project research team, Maria Abreu, Paul Beck, Charles Bernstein, Kenneth Croitoru, Levinus Dieleman, Brian Feagan, Anne Griffiths, David Guttman, Kevan Jacobson, Gilaad Kaplan, Denis O. Krause, Karen Madsen, John Marshall, Paul Moayyedi, Ernest Seidman, Mark Silverberg, Andy Stadnyk, A. Hillary Steinhart, Michael Surette, Dan Turner, Thomas Walters, Bruce Vallance, Guy Aumais, Alain Bitton, Maria Cino, Jeff Critch, Lee Denson, Colette Deslandres, Wael El-Matary, Hans Herfarth, Peter Higgins, Hien Huynh, Jeff Hyams, David Mack, Jerry McGrath, Anthony Otley, Remo Panancionne, Robert Baldassano, Anne M. Griffiths, Charlotte Hedin, Seamus Hussey, Hien Hyams, David Keljo, David Kevans, Charlie Lees, Sanjay Murthy, Remo Panaccione, Nimisha Parekh, Sophie Plamondon, Graham Radford-Smith, Mark Ropeleski, Joel Rosh, David Rubin, Michael Schultz, Corey Siegel, Scott Snapper, Andrew D. Paterson, Kenneth Croitoru

**Affiliations:** 1 Department of Medicine, University of Toronto, Toronto, ON Canada; 2 Zane Cohen Centre for Digestive Diseases, Mount Sinai Hospital, 600 University Avenue Room 437, Toronto, Ontario M5G 1X5 Canada; 3 Lunenfeld-Tanenbaum Research Institute, Sinai Health System, Toronto, Ontario Canada; 4 Division of Biostatistics, Dalla Lana School of Public Health, University of Toronto, Toronto, Ontario Canada; 5 Department of Cell & Systems Biology, University of Toronto, Toronto, Ontario Canada; 6 Centre for the Analysis of Genome Evolution & Function, University of Toronto, Toronto, Ontario Canada; 7 Division of Gastroenterology, Hepatology and Nutrition, Department of Paediatrics, The Hospital for Sick Children, Toronto, Ontario Canada; 8 Department of Medicine, McMaster University, Hamilton, Ontario Canada; 9 Inflammatory Bowel Disease Clinic, Division of Gastroenterology and Hepatology of Gastroenterology, University of Calgary, Calgary, Alberta Canada; 10 Department of Pediatrics, University of Alberta, Edmonton, Alberta Canada; 11 Hôpital Maisonneuve-Rosemont, Department of Medicine, Montreal University, Montreal, Quebec Canada; 12 Division of Gastroenterology and CEGIIR, Department of Medicine, University of Alberta, Edmonton, Alberta Canada; 13 Department of pediatric GI, Shaare Zedek Medical Center, 91031 Jerusalem, Israel; 14 Division of Epidemiology, Dalla Lana School of Public Health, University of Toronto, Toronto, Ontario Canada; 15 Genetics and Genome Biology, The Hospital for Sick Children Research Institute, The Hospital for Sick Children, Toronto, Ontario Canada

**Keywords:** Fecal microbiota, Healthy human, Microbiome, rs2066844, rs2066845, rs2066847, *NOD2*, Inflammatory bowel disease

## Abstract

**Background:**

Genetic analyses have identified many variants associated with the risk of inflammatory bowel disease (IBD) development. Among these variants, the ones located within the *NOD2* gene have the highest odds ratio of all IBD genetic risk variants. Also, patients with Crohn’s disease (CD) have been shown to have an altered gut microbiome, which might be a reflection of inflammation itself or an effect of other parameters that contribute to the risk of the disease. Since NOD2 is an intracellular pattern recognition receptor that senses bacterial peptidoglycan in the cytosol and stimulates the host immune response (Al Nabhani et al., PLoS Pathog 13:e1006177, 2017), it is hypothesized that *NOD2* variants represent perfect candidates for influencing host-microbiome interactions. We hypothesized that *NOD2* risk variants affect the microbiome composition of healthy first degree relative (FDR) of CD patients and thus potentially contribute to an altered microbiome state before disease onset.

**Methods:**

Based on this, we studied a large cohort of 1546 healthy FDR of CD patients and performed a focused analysis of the association of three major CD SNPs in the coding region of the *NOD2* gene, which are known to confer a 15–40-fold increased risk of developing CD in homozygous or compound heterozygous individuals.

**Results:**

Our results show that carriers of the C allele at rs2066845 was significantly associated with an increase in relative abundance in the fecal bacterial family Erysipelotrichaceae.

**Conclusions:**

This result suggests that *NOD2* polymorphisms contribute to fecal microbiome composition in asymptomatic individuals. Whether this modulation of the microbiome influences the future development of CD remains to be assessed.

## Background

Crohn’s disease (CD) is a complex disease with a significant public health impact [1]. Several factors are known to contribute to the risk of developing CD, but the exact cause(s) remains to be identified. It is generally thought that genetic and environmental factors play a crucial role in the development of CD. Also, alterations in the gut microbiome are described in patients with established CD [2]. *NOD2* was the first susceptibility gene discovered for CD [3]. Since this discovery, genome-wide association studies (GWAs) have allowed the identification of many other genetic variants associated with CD [4], including several additional variants located in the *NOD2* locus [4]. More precisely, three common variants located in the *NOD2* leucine-rich repeat domain have been identified in the European population. These are R702W, G908R resulting from two cis-mutations in rs2066844, rs2066845 and L1007fs, a frameshift mutation identified by rs2066847 insC, respectively [5, 6]. Individuals who are homozygous or compound heterozygous for these mutations in *NOD2* have a 15–40-fold increased risk of developing CD [5, 6].

Previous studies attempted to determine if *NOD2* genotype is associated with any alteration in the composition of the intestinal microbiome in CD case-control studies. However, those studies fail to distinguish between the possibilities that microbial changes are the result of inflammation present in these patients with CD or drug therapies [7]. Several studies have performed such analysis using a GWAs approach in healthy individuals, but all failed to identify robust association looking at *NOD2* genotype association with microbial composition [8–10]. The conservative threshold for association significance used in GWAs studies (*p*-value < 5 × 10^− 8^), may explain the absence of association. Alternatively, healthy individuals from the general population may have a relatively lower abundance of *NOD2* variants associated with CD. For these two reasons, we addressed in a targeted fashion whether *NOD2* CD risk alleles are associated with microbial composition in healthy first-degree relatives (FDR) of CD patients known to be genetically at higher risk to develop CD as compared to general population [11, 12]. We thus evaluated in a targeted fashion the association between three *NOD2* CD risk variants known to have the largest effect size for CD (rs2066844, rs2066845 and rs2066847) with the fecal microbial composition in a large cohort of 1546 healthy FDRs of CD patients.

## Methods

### Patient recruitment

A total of 1546 subjects were recruited as part of the Crohn’s and Colitis Canada Genetic Environmental Microbial (CCC-GEM) project between the years 2008 and 2017. The study was approved by the Mount Sinai Hospital Research Ethics Board (Toronto - Managing center) and each participating recruitment center. All the participants or their guardians provided written consent before their enrollment. Participants provided blood and a stool sample for genetic and microbiome analysis, respectively.

### Genotyping

DNA extraction from peripheral blood mononuclear cells (PBMC) was performed using the Gentra Puregene Blood Kit (Qiagen, CA, USA). Genomic DNA was quantified using Nanodrop®, and then dilutions were prepared at concentrations of 20 ng/μl and aliquoted into 96 well reaction plates. SNPs genotyping was performed using Illumina HumanCoreExome chip and ImmunoChip (Illumina, Inc. San Diego, CA) following the provider’s recommendations as previously described [8].

### Genome imputation

Genotype imputation was performed on a merged HumanCoreEXOME chip and ImmunoChip hard genotype data. We performed two imputations, the first one based on the Haplotype Research Consortium (HRC) panel, and the second based on the 1000 Genomes imputation panel of October 2014. The former was performed by directly uploading the hard genotype to the HRC website (http://www.haplotype-reference-consortium.org/), while for the latter, IMPUTE2 (v. 2.3.0) [13, 14] was used. Markers were imputed in 1 M bp intervals with 750 K bp flanking regions with pre-phased haplotypes. Imputation results were quality controlled for minor allele frequency (MAF > 1%), missingness (< 5%), imputation certainty (> 0.4), and imputation information (> 0.9). The three *NOD2* SNPs were identified based on their coordinates in the human genome build 37. The two SNPs rs2066844, rs2066845 were captured from the HRC imputation, whereas the rs2066847 was present as a hard-genotyped SNP in HumanCoreEXOME chip. PLINK v1.90b3.38 was used to extract the SNPs and to calculate their MAF [15].

### Taxonomic profiling of the gut microbiota

Stool samples were collected in FB Commode Specimen Collector (Fisher Scientific, Waltham, MA) and put into polypropylene vials (Starplex Scientific Inc., Etobicoke, ON) and frozen prior their shipping to the local study sites where they were stored at − 80 °C. DNA extraction from fecal bacteria was performed using the QIAamp DNA Stool Mini Kit (Qiagen, Hilden, Germany). We added a step to the manufacturer’s recommendations, which consists of physical disruption of the bacterial cell wall using ceramic beads. The V4 hypervariable region of bacterial 16S ribosomal RNA (16S rRNA) was sequenced in paired-end mode (2 × 150 base pair) using MiSeq platform (Illumina Inc., San Diego, CA, USA). Paired reads were assembled using fastq-join software (ea-utils suite v1.1.2.537), resulting in 250 base pairs amplicons. The latter were then processed using the quantitative insights into microbial ecology (QIIME v1.9.0) pipeline using the default parameters. Operational taxonomic units (OTUs) were clustered at 97% sequence similarity against Greengenes database v.13_8 using a closed reference approach. Samples with less than 10,000 reads after quality filtering were removed from the analysis. Alpha diversity was expressed using the Shannon index after rarefaction at a depth of 10,000 reads per sample. 16S sequences are available by the study accession number PRJEB14839 [8].

### Statistical considerations

To assess the association between the *NOD2* polymorphisms and the fecal microbiome, we used two approaches. In the first approach, we evaluated the individual association of each of the three *NOD2* risk SNPs with the microbiome. In the second approach, we stratified the cohort based on the presence of at least one *NOD2* risk variant. Hence we had a new variable with two categories: subjects with at least one mutation (rs2066844, and/or rs2066845, and/or rs2066847) and subjects with no mutations.

In the two approaches, we tested first the association of the variables with the global composition of the microbiome at the genus level using permutational multivariate analysis of variance (PERMANOVA) on an OTU table rarefied to 10,000 reads and using a Bray-Curtis distance matrix. Then, the variables (*NOD2* polymorphism and presence of at least one variant) were tested for their association with every member of the different taxonomy levels from phylum to genus using a two-part log-normal model as previously described [8, 16]. The model was applied to a non-rarefied OTU table and corrected for age, sex and the total number of reads per sample. All statistical analyses were performed using R (v3.3.3). PERMANOVA was performed using the Vegan package (V2.4–5), whereas the *geeglm* function of the geepack package (v1.2.0–2) was used to carry out the two-part log-normal model on each bacterial taxa.

The correction of multiple testing was performed by calculating the effective number of uncorrelated taxa using the approach described by Li and Ji [17]. Our taxonomy table contained 263 taxa from phylum to genus level. The application of the Li and Ji [17] method allowed us to identify 132 independent variables, bringing the raw *p*-value significance threshold to 3.8 × 10^− 4^.

## Results

### Frequency of *NOD2* risk variants in healthy FDR of CD patients

A cohort of 1546 asymptomatic FDRs of CD patients recruited between 2008 and 2017 as part of the Crohn’s and Colitis Canada Genetic Environmental Microbial (CCC-GEM) project. All study subjects were of Caucasian descent with a mean age ± standard deviation of 19.4 ± 7.9 [6-35] were included in this study including 848 Female (See methods section). We calculated the MAF of the rs2066844 (R702W), rs2066845 (G908R) and rs2066847 (l1007fs) in our population of healthy FDRs of CD patients and compared this to those recorded in the 1000 genome project (self-reported healthy individuals) (see Table 1). The MAF of each of the three *NOD2* risk SNPs were numerically higher in our cohort than reported in the 1000 genome project general healthy population. This increase in SNP frequency suggests that our cohort is enriched in *NOD2* risk SNPs.

**Table 1:** Minor allele frequency of *NOD2* risk SNPs in healthy FDR of CD patients in comparison to the MAF in the 1000 genome project

SNPs	MAF in healthy FDR of CD	MAF in the 1000 genomes project
rs2066844	8.9%	1%
rs2066845	3.1%	< 1%
rs2066847	4.2%	< 1%

MAF: Minor allele frequency, SNPs: single nucleotide polymorphisms. MAF from the 1000 genomes project was obtained using Ensembl database

### Association of the individual *NOD2* risk SNPs with the microbial composition and diversity of the microbiome in FDRs

To understand the associations between the three individual *NOD2* variants and the significance in clustering patterns of the microbiome composition, we performed a PERMANOVA analysis; this showed no significant association (Table 2). Similarly, the group with at least one out of the three *NOD2* variants compared with individuals without any *NOD2* variant showed no significant difference in clustering patterns of stool microbiome composition. The alpha diversity, as represented by the Shannon index, also showed no significant association with each of the individual *NOD2* variants, rs2066844, rs2066845 or rs2066847, respectively. The group with at least one mutation in the three *NOD2* risk SNPs compared to the group with no variants also showed no significant difference in alpha diversity.

**Table 2:** Association between the three *NOD2* polymorphisms and the clustering patterns of the fecal microbiome composition

Variants	PERMANOVA *p* value
rs2066844	0.44
rs2066845	0.07
rs2066847	0.10
At least one *NOD2* mutation^a^	0.75

^a^ in rs2066844 and/or rs2066845 and/or rs2066847

### Association between *NOD2* polymorphisms and specific fecal bacterial taxa and diversity

**Fig. 1:**
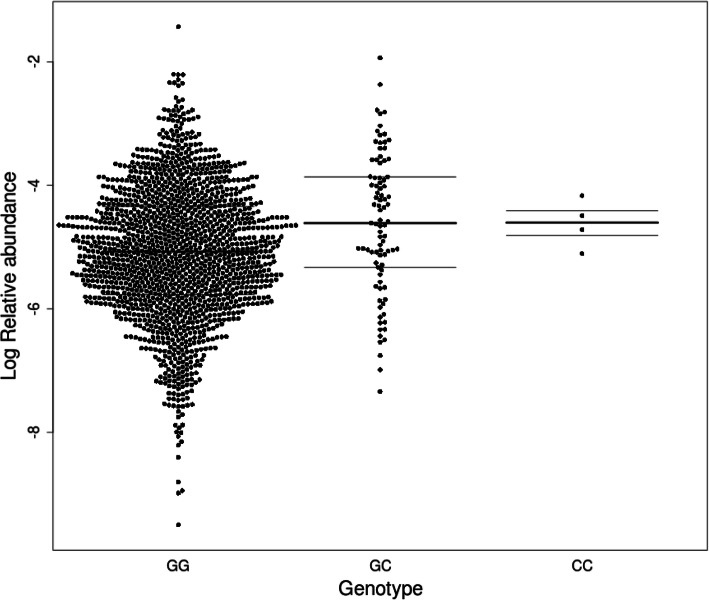
Erysipelotrichaceae family is associated with rs2066845 genotype in 1546 healthy subjects. The x-axis corresponds to the rs2066845 genotype in the cohort. The y-axis corresponds to the log-transformed relative abundance of Erysipelotrichaceae. Each dot represents the value for a given individual. The lines represent the first, second, and third quartiles. Carrier of the C allele are enriched in the relative abundance of Erysipelotrichaceae (*p*-value = 7.14 × 10^− 5^)

We assessed the association of each of the three *NOD2* variants with each fecal bacterial taxon from phylum to genus level of the taxonomy. The rs2066845 was significantly associated with the family *Erysipelotrichaceae* (estimate ± standard deviation; 0.39 ± 0.10, *p*-value = 7.14 × 10^− 5^) (Fig. 1). The abundance of this taxon was significantly increased in the groups with at least one mutation in rs2066845 (the heterozygote and the minor homozygote) compared to groups with the null mutation. However, none of the genera of the *Erysipelotrichaceae* family appeared to significantly contribute to the effect observed at the family taxonomic level (Supplementary Table 1). No significant associations were observed for rs2066844 or rs2066847 with microbiome taxa (Supplementary Tables 2 and 3, respectively). When assessing the effect of binning the three *NOD2* variants, no significant associations were observed between the presence of at least one mutation within the three *NOD2* variants with any of the individual taxa (Supplementary Table 4).

## Discussion

The *NOD2* gene was the first found to be directly associated with CD risk through three main mutations occurring in the leucine-rich repeat region of the gene. Since this gene is a pattern recognition receptor and is involved in host-microbe interactions, a few studies have shown that *NOD2* polymorphisms are involved in shaping the microbiome in IBD patients [18–21]. However, most of these studies were case-control studies, and as such, the inflammation process can affect microbiome composition regardless of the *NOD2* genotype [19–21]. Our study cohort of healthy FDRs of CD patients permits to minimize some of these potential confounding factors as they are asymptomatic, do not take any anti-inflammatory or anti-bacterial medication, while having a high genetic risk of developing CD and carrying a higher frequency of CD associated mutations. As such, we assessed the association of three major *NOD2* mutations with the composition of the gut microbiome in this cohort. We found that this cohort was enriched in *NOD2* variants as reflected by higher MAFs of the CD risk SNPs, rs2066844 (R702W), rs2066845 (G908R), and rs2066847 (l1007fs) as compared to the general population (Table 2) confirming the findings of our previous study of healthy first-degree relatives [22]. Hence this cohort provides a higher prevalence of the *NOD2* polymorphisms and thus is more powered than cohorts of healthy individuals from the general population in order to assess the potential association of *NOD2* SNPs with gut microbiome composition in healthy individuals.

Our results showed that one of the *NOD2* risk allele (rs2066845) is associated with the increased relative abundance of *Erysipelotrichaceae* family. Little is known regarding *Erysipelotrichaceae* family in terms of disease associations [23]. There is data to suggest that *Erysipelotrichaceae* is associated with metabolic disorders in humans [23]. Another study showed that species within *Erysipelotrichaceae* family are positively linked to inflammation in patients who had chronic HIV infection [19]. This taxon was decreased in early CD onset [20], and patients who experienced recurrence of CD had significantly lower levels of *Erysipelotrichaceae* [21]. Studies have shown inconsistent alterations in *Erysipelotrichaceae* family relative abundance in patients with established CD [7, 20, 21, 23]. In a recent report, *Erysipelotrichaceae* family was found to be increased in patients with CD patients with ileal disease compared to those with ileocolonic disease [24], which may be consistent with the fact that *NOD2* is strongly associated with ileal CD [25]. Also, this taxon was found to be highly coated by IgA relative to other members of the gut microbiota in IBD patients [26]. It includes strictly anaerobic or aerotolerant organisms, and members of the Erysipelotrichaceae family can produce palmitic, stearic and oleic acid, fatty acids predominantly [27]. Over accumulation of tissue palmitic acid results in dyslipidemia, hyperglycemia, increased ectopic fat accumulation, increased inflammatory tone via toll-like receptor 4 [28] as well as pro-inflammatory adipokines mRNA expression in macrophages [29]. Also, stearic acid was shown to stimulate pro-inflammatory cytokine production [30]. It remained to be shown how the changes in this taxon contribute to the risk of CD in this population.

The other two *NOD2* risk variants (rs2066844 and rs2066847) showed no significant associations with the fecal bacterial composition. A previous study detected an association of *NOD2* risk allele dosages (comprised of combined rs104895431, rs104895467, rs2066844, rs2066845, rs5743277, rs5743293) with an increase of Enterobacteriaceae from biopsy sample in a cohort of 474 IBD affected patients [31]. However, the altered microbiome described in IBD patients is characterized by an increase in Proteobacteria species such as *Escherichia coli* (a species belonging to Enterobacteriaceae family) [2, 32]. Thus the increase of Enterobacteriaceae previously observed might be related to inflammation itself and not necessarily *NOD2* genotype. Other studies of large cohorts of healthy individuals have failed to show any significant association of *NOD2* genotype with microbiome composition [7–10, 33]. In these GWAS, only one study comprised of 1514 individuals showed that the *NOD2* locus was associated with the enterobactin biosynthesis pathway, which is highly correlated with *Escherichia coli* abundance [10]. Our analysis showed that all the three tested *NOD2* variants had a consistent direction of the association for which *NOD2* variants had an increased Enterobacteriaceae despite not being significant. Thus the association of Enterobacteriaceae and *NOD2* locus polymorphisms remains unclear.

The association between host genetics with microbial composition might be better detected in biopsy samples compared to fecal samples. In fact, in a study comparing the tissue associated bacteria in the ileum to the fecal bacteria in CD patients carrying the rs2066847 polymorphism or not showed that significant differences in microbial composition were only observed in the biopsy microbiome [34]. Recent GWAs studies of IBD subjects report that *NOD2* variants are strongly associated in particular with ileal CD compared to colonic CD [25]. Thus, this may, in part, explain the weaker signal observed in fecal samples compared to biopsy samples in the ileum [35, 36].

## Conclusion

In conclusion, this study is one of the largest studies of healthy subjects examining the specific association of the *NOD2* genotype with the healthy human gut microbiota. We found that the *NOD2* genotype has no significant influence on the overall composition of stool microbiota, suggesting that *NOD2* polymorphism is not a major determinant of the overall composition of the microbiome in this healthy at-risk population. However, in our targeted analysis of the microbiota, we found that rs2066845 is significantly associated with an increased relative abundance of the Erysipelotrichaceae family. It remains possible that the effect of *NOD2* polymorphisms on microbiome composition are limited under stable homeostatic conditions, and might be more enhanced after the disease onset, i.e. in the presence of active inflammation [31, 34, 37], or may be related to a specific location such as ileal CD. It remains possible that increased risk of developing CD contributed by *NOD2* genotype is in part related to alteration of particular taxa such as Erysipelotrichaceae family in healthy individuals.

## Supplementary information

**Additional file 1 Supplementary Table 1**. Association of rs2066845 with bacterial taxa in the GEM cohort. **Supplementary Table 2**. Association of rs2066844 with bacterial taxa in the GEM cohort. **Supplementary Table 3.** Association of rs2066847 with bacterial taxa in the GEM cohort. **Supplementary Table 4.** Cumulative *NOD2* variants association with bacterial taxa in the GEM cohort.

## Data Availability

16S sequences are available by the study accession number PRJEB14839 (NCBI BioProject database); ENA-SUBMISSION: ERA675979 [9]. Requests for raw and analyzed data should follow the instruction given at http://www.gemproject.ca/data-access/. All submissions will be reviewed by the GEM Project Operating Committee to ensure that the requested samples/data will not interfere in any way with the intended GEM Project analysis of the nested cohort as per the original GEM Project Study Design and is not a duplication of analysis already ongoing. Those proposals meeting this evaluation will be distributed to all members of the GEM Project Steering Committee (GPSC) for review and open discussion. This review will focus on the global scientific merit of the proposal. This review will assess the basic scientific merit and the availability of requested samples and data, ensuring there is no compromise of the original intent of the GEM project. It would be of value to contact a member of the Steering Committee who could help sponsor your application. Those projects achieving majority vote of approval at the GPSC will be informed that the GEM Project will provide a letter of support stating that the requested samples or data will be made available to the applicants once the applicant receives funding from a granting agency that applies an independent peer review process to the proposal. The criteria to be used for review of all submissions will include the “scientific relevance” of the proposal and the judged availability of biological material requested. The budget to be requested from a funding agency, must allow for any expenses in processing samples or in setting up the appropriate queries of the database. The intent is to allow sufficient time for applicants to consider submission for funding opportunities.

## Abbreviations

CD: Crohn’s disease
IBD: Inflammatory bowel disease
FDR: Healthy first degree relative
GWAs: Genome-wide association studies
CCC-GEM: Crohn’s and Colitis Canada Genetic Environmental Microbial
PBMC: Peripheral blood mononuclear cells
PERMANOVA: Permutational multivariate analysis of variance
HRC: Haplotype Research Consortium
OTUs: Operational taxonomic units
MAF: Minor allele frequency
SNP: Single nucleotide polymorphisms
NOD2: Nucleotide-binding oligomerization domain-containing protein 2
16S rRNA: 16S ribosomal RNA
QIIME: Quantitative insights into microbial ecology
